# Surgeon at Work: Hand-Assisted Laparoscopic Construction of Gastric Conduit for Thoracic Esophageal Cancer

**DOI:** 10.1155/DTE.7.75

**Published:** 2001

**Authors:** Tatsuya Aoki, Akihiko Tsuchida, Yoshiaki Osaka, Yu Takagi, Motoo Shinohara, Ryosuke Okada, Hidenori Tomioka, Yasuhisa Koyanagi

**Affiliations:** Department of Surgery Tokyo Medical University 6-7-1 Nishi-Shinjuku Shinjuku-Ku Tokyo Japan

## Abstract

A method for hand-assisted laparoscopic construction of gastric conduit for thoracic esophageal cancer was developed. Since this endoscopic surgical procedure is less invasive than open surgery, it contributes to improvement of post-operative respiratory functions and reduces respiratory complications. What distinguishes our surgical procedure is that unlike methods described in previous reports, it begins with treatment of the left gastroepiploic vessels at the height of the inferior edge of the spleen, followed by dissection from the esophageal hiatus to the lesser curvature and then dissection and excision of left gastric arteries and veins. Finally, the exposed esophagus and stomach are drawn outside the body and the right gastroepiploic blood vessels are preserved, followed by dissection of the greater omentum. This approach to gastric conduit construction was undertaken in 6 patients and the mean operating time was 123 minutes. Although in the first 3 of these patients the operating time was 150 minutes or more, the time required shortened to around 90 minutes for each of the last 3 cases, as the procedure was mastered. In each case, the volume of intraoperative hemorrhage did not exceed 50 ml.


*Diagnostic and Therapeutic Endoscopy*, Vol. 7, pp. 75–81
Reprints available directly from the publisher
Photocopying permitted by license only

© 2001 OPA (Overseas Publishers Association) N.V.
Published by license under
the Harwood Academic Publishers imprint,
part of Gordon and Breach Publishing,
a member of the Taylor & Francis Group.

*Surgeon at Work*

### Hand-Assisted Laparoscopic Construction of Gastric Conduit for Thoracic Esophageal Cancer

TATSUYA AOKI*, AKIHIKO TSUCHIDA, YOSHIAKI OSAKA, YU TAKAGI, MOTOO SHINOHARA, RYOSUKE OKADA, HIDENORI TOMIOKA and YASUHISA KOYANAGI

*Department of Surgery, Tokyo Medical University, 6-7-1 Nishi-Shinjuku, Shinjuku-Ku, Tokyo, Japan*

*(Received 7 December 2000; In final form 30 January 2001)*

**A method for hand-assisted laparoscopic construction of gastric conduit for thoracic esophageal cancer was developed. Since this endoscopic surgical procedure is less invasive than open surgery, it contributes to improvement of post-operative respiratory functions and reduces respiratory complications. What distinguishes our surgical procedure is that unlike methods described in previous reports, it begins with treatment of the left gastro-epiploic vessels at the height of the inferior edge of the spleen, followed by dissection from the esophageal hiatus to the lesser curvature and then dissection and excision of left gastric arteries and veins. Finally, the exposed esophagus and stomach are drawn outside the body and the right gastroepiploic blood vessels are preserved, followed by dissection of the greater omentum. This approach to gastric conduit construction was undertaken in 6 patients and the mean operating time was 123 minutes. Although in the first 3 of these patients the operating time was 150 minutes or more, the time required shortened to around 90 minutes for each of the last 3 cases, as the procedure was mastered. In each case, the volume of intraoperative hemorrhage did not exceed 50 ml.**

*Keywords*: Esophageal cancer, Gastric conduit, Hand-assisted laparoscopic surgery

### INTRODUCTION

Advances in endoscopic surgery have brought many benefits to patients, including less post-operative pain, shorter hospital stay, and cosmetic advantages. In recent years, endoscopic surgery has been applied for esophageal cancer and thoracoscopic esophagectomy has been performed [1–8]. Nevertheless, there are only a few reports of laparoscopic construction of gastric conduit for esophageal cancer, and the procedure has not yet been finally established [9–11]. Consequently, we report our surgical procedure for hand-assisted laparoscopic construction of a gastric conduit.

* Corresponding author. Tel.: 81-3-3342-6111, ext. 5835. Fax: 81-3-3340-4575.

### PATIENTS AND METHODS

### Patients

During the period of April 2000 to November 2000, 16 patients with thoracic esophageal cancer were surgically treated at the Department of Surgery, Tokyo Medical University Hospital. Ten of sixteen patients had apparent lymph node metastasis in the abdominal cavity by the preoperative diagnostic image, which were excluded to apply the laparoscopic procedure. Therefore, 6 cases were selected for this method. All 6 patients could tolerate general anesthesia without severe organ dysfunction. Their tumors were located from the neck to the middle thoracic esophagus without any severe adhesion due to previous surgery or inflammation in the abdominal cavity. According to the UICC classification, the clinicopathological data is summarized in Table I.

### Operative Procedure

Open or thoracoscopic surgery was performed prior to maneuver in the abdominal cavity. The esophagus was resected in the upper thorax and wound was closed. Then, the patient was switched to a supine position and both legs were parted. The operator stood in between the legs and two assistants stood on either side of patient. The abdomen was opened by making a small incision of about 7.0 cm from a point 3 cm below the xiphoid process at the anterior median of the epigastrium. A Lapdisc® (Hakko Medical Co., Tokyo, Japan) was attached to the same region and the operator inserted his left hand into the abdominal cavity through it. Then, a laparoscope was inserted below the umbilicus. Insufflation was carried out at a pressure of 8–10 mm Hg, and 12-mm trocars were

TABLE I Clinicopathological data of 6 patients

| No. | Age | Sex | Location | Histology | T | N | M | *p*-Stage |
|---|---|---|---|---|---|---|---|---|
| 1 | 44 | male | UT | G2 | 3 | 1 | la | IVa |
| 2 | 54 | male | MT | G2 | 1 | 0 | 0 | I |
| 3 | 48 | male | MT | G2 | 3 | 1 | 0 | III |
| 4 | 63 | male | MT | G1 | 2 | 1 | 0 | IIb |
| 5 | 58 | male | UT | G2 | 3 | 1 | 0 | III |
| 6 | 50 | male | MT | G3 | 2 | 1 | 0 | IIb |

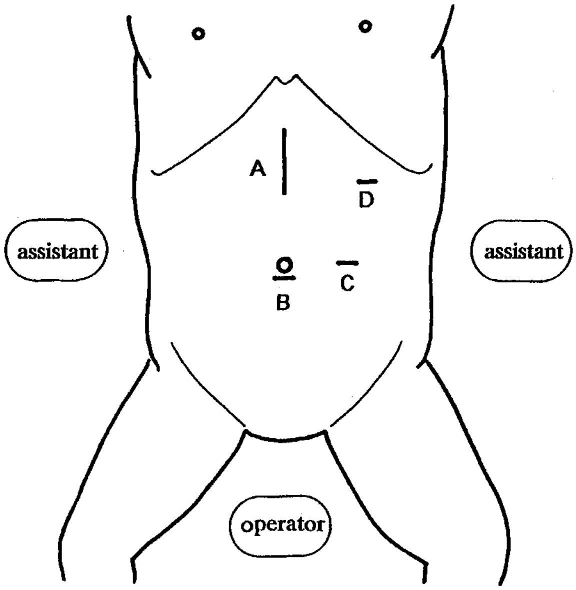

FIGURE 1 The operative position of surgeons, skin incision, and trocar sites. A: 7 cm skin incision for the Lapdisk®; B, C, D: 12 mm trocars.

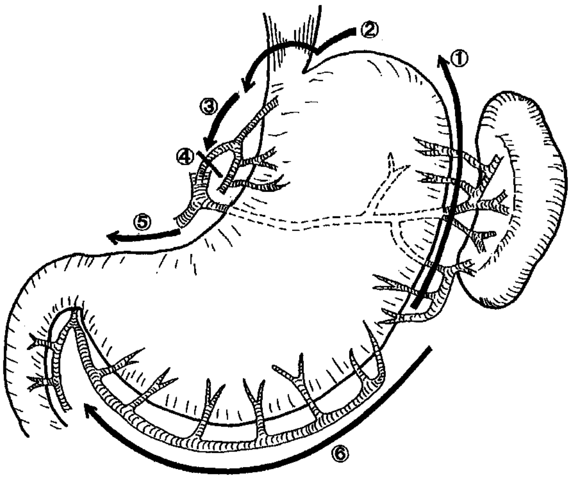

FIGURE 2 Operative procedure: ① dissection of the left gastroepiploic and short gastric vessels; ② dissection of the surrounding esophageal hiatus; ③ dissection of the surrounding lesser curvature in the upper stomach; ④ dissection of the left gastric vessels; ⑤ dissection of the surrounding lesser curvature toward the pylorus; ⑥ dissection of the greater curvature toward the pylorus.

inserted to the left of the umbilicus and over the midline of the left costal arch, respectively (Fig. 1).

Our surgical procedure is shown in Fig. 2. While retracting the stomach to the cephalic side with the operator's left hand, the positional relationship between the greater omentum and the spleen should be determined. The operator holds Laparoscopic Coagulating Shears® (LCS; Johnson & Johnson Medical, Cincinnati, USA) connected to a Harmonic-Scalpel® and uses it to dissect the greater omentum at a height nearly equal to that of the splenic inferior edge. With Babcock forceps, an assistant retracts the greater omentum or transverse colon at the caudal side. From this region, the short gastric arteries and veins are coagulated and cut away by LCS along the greater curvature until the esophageal hiatus (Fig. 3). Then, while retracting the left lobe of the liver to the cephalic side using the back of the left hand, the operator completes dissection around the esophageal hiatus (Fig. 4). The resected esophagus is withdrawn into the abdominal cavity with the left hand. While retracting the esophagus and the upper part of the stomach to the caudal side with the left hand, dissection around the lesser curvature is performed. Subsequently, dissection is continued around the left gastric arteries and veins, and these vessels are exposed and cut using an Endocutter® (ETS Flex 35; Johnson & Johnson Medical) (Fig. 5). In addition, the lesser omentum is dissected in the direction of the duodenum. After completion of insufflation, the exposed esophagus and stomach are withdrawn to outside the body with the left hand. While preserving the right gastroepiploic vessels, the remaining greater omentum is dissected in the direction of the duodenum. Finally, using a Linear Cutter® (Johnson & Johnson Medical), the stomach is partly cut at the lesser curvature to construct a gastric conduit.

### RESULTS

The surgical procedure was successfully completed in all 6 patients, none of whom required conversion to open surgery. The operating times in each

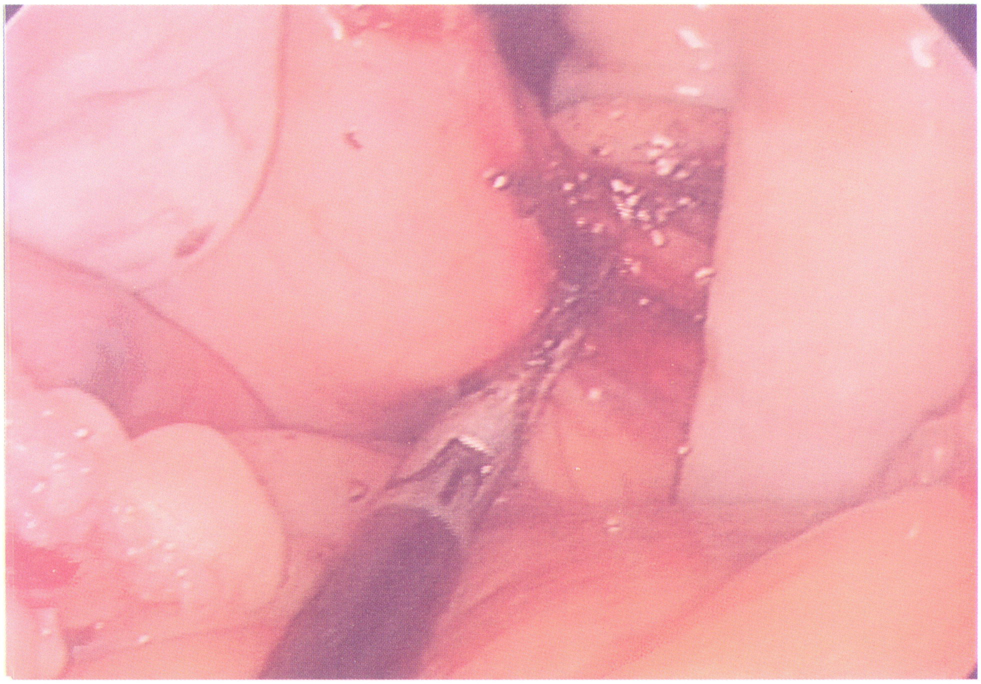

FIGURE 3 Dissection of short gastric vessels along the greater curvature using LCS®.

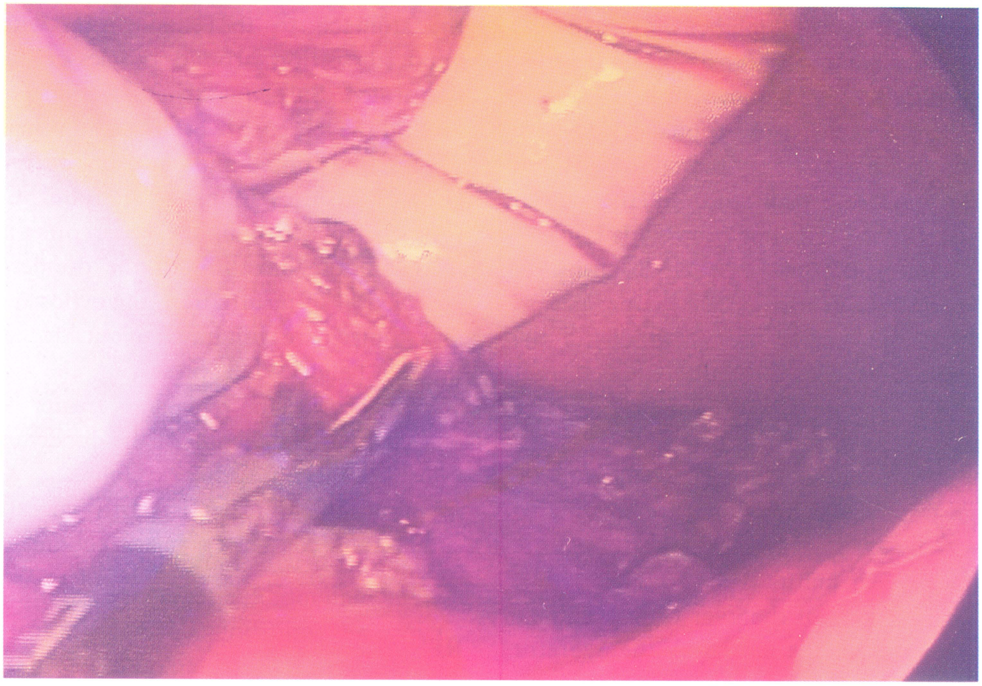

FIGURE 4 Dissection of the right side crus of diaphragm using LCS®.

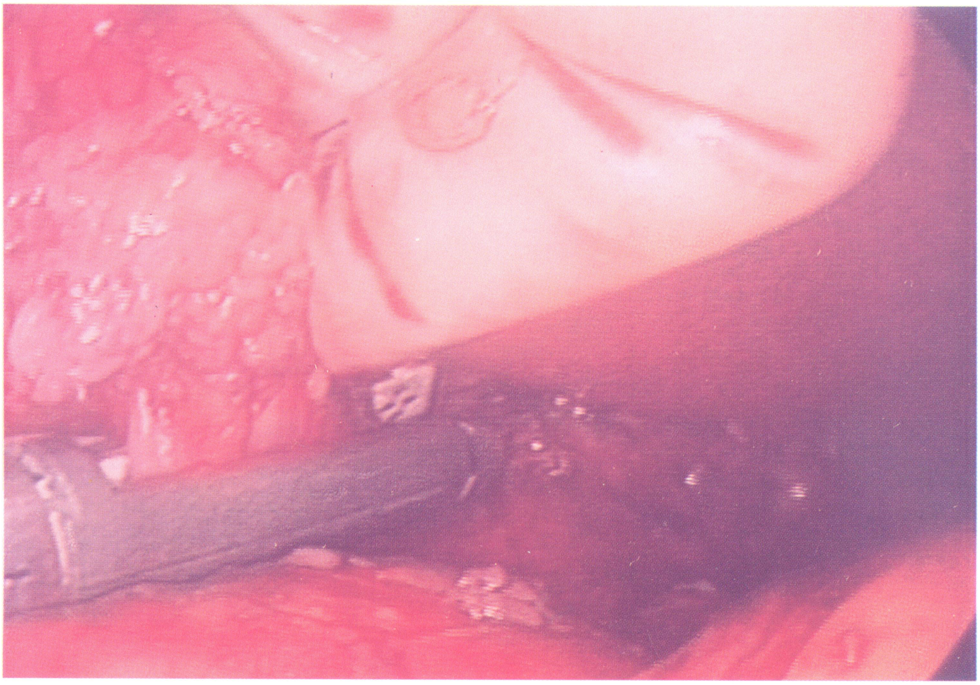

FIGURE 5 Cutting of the left gastric artery and veins using an Endocutter®.

patient were as follows; 165 minutes, 150 minutes, 150 minutes, 90 minutes, 90 minutes and 90 minutes (mean: 123 minutes) in chronological order. The volume of intraoperative hemorrhage did not exceed 50 ml in any case.

### DISCUSSION

In recent years, endoscopic surgery has spread remarkably, and its application has been expanding in many surgical fields. In open esophagectomy, a high incidence of respiratory morbidity (from 6% to 33%) has been reported [3,12]. To reduce these complications, thoracoscopic esophagectomy has attracted interest as a less invasive procedure [1–8]. Reports of transhiatal blunt dissection of the esophagus in laparoscopic surgery without thoracotomy also have been increasing [13–15]. With the development of these surgical procedures, Inoue *et al.* first succeeded in performing laparoscopic gastric reconstruction with thoracoscopic esophageal resection [11]. Although the laparoscopic approach in upper abdominal surgery preserves respiratory function [16,17], there are only several reports of laparoscopic construction of gastric conduit [9,10,15]. This is probably because the totally laparoscopic approach is technically very difficult to complete. However, the hand-assisted laparoscopic surgery (HALS) developed by Kusminsky *et al.* is a new concept for surgeons [18]. It has been applied in various abdominal surgical procedures, including transhiatal blunt dissection of the esophagus, because it allows the operator, by inserting one hand into the abdominal cavity, to perform detailed manipulations which could not be done through the totally laparoscopic method [19–21]. The advantage of this approach is that the organ can be easily grasped, retracted and dissected by inserting a hand directly into the abdominal cavity. Moreover, surgical manipulations can be carried out quickly and accurately through hand touch. Accordingly, it can be applied for the advanced cancer operations requiring lymph node dissection. Yoshida *et al.* commented on the following differences between HALS and laparoscopic assisted surgery (LAS): (1) the operator's hand can be used as one set of endoscopic forceps, (2) whereas insufflation is not generally used in LAS, the entire procedure is essentially conducted under endoscopy and insufflation in HALS, and (3) in LAS, a small incision is made for mini-laparotomy, but in HALS a large hole is made through which the hand can be inserted. Consequently, whereas incision can be made directly over target organs in LAS, it must be made at a point distant from the target organ to preserve hand mobility in the abdominal cavity in HALS [22]. The position of small incision for introduction of left hand is still a matter of discussion. Yoshida *et al.* placed an oblique incision in the right lower abdomen [22], whereas Ozawa *et al.* made a transverse incision in the upper median abdomen [23]. On the contrary, we performed a median incision in the upper abdomen according to the same method as Inoue *et al.* [11]. An upper median incision has several advantages as follows:

1. It takes less damage to muscle and fascia,
2. It is easy to manipulate the dissection around esophageal hiatus, because the left hand is closer to it in comparison with other incisions, and
3. In cases of operative emergency such as massive bleeding or iatrogenic organ injury, it is easy to manage it by the prolongation of median incision.

Previously reported surgical procedures for gastric conduit construction were as follows. The gastrohepatic ligament was first dissected, and the crus of the diaphragm was subsequently, exposed on the right side of the esophagus. Then, dissection was continued along the greater curvature, and the short gastric vessels were ligated and dissected [9–11]. In contrast, our procedure differs completely from this conventional approach. Dissection is first performed along the greater curvature from a height equivalent to the splenic inferior edge in the direction of esophagus, followed by dissection around the esophagus and crus of diaphragm. After the esophagus is withdrawn into the abdominal cavity, the gastrohepatic ligament is dissected. The advantage of this method is that the surgical procedure is easy to perform. Since the trocars are usually placed at the caudal side from the greater curvature, dissection of the lesser curvature, which is in a tangential direction, can sometimes be difficult. In our approach, dissection around the lesser curvature was easy to perform, because it could be visualized from the dorsal side of the stomach and manipulations with the Harmonic Scalpel or forceps could be simplified by withdrawal of the resected esophagus to the abdominal cavity. Inoue *et al.* pointed out that when thoracic manipulation had to be performed prior to abdominal surgery, insufflation gas flowed into the thoracic cavity immediately after the esophagus was withdrawn into the abdominal cavity, and operation in the abdominal cavity under insufflation was impossible [11]. In our experience, although a slight volume of insufflation gas flowed into the thoracic cavity when the esophagus was withdrawn, there was no subsequent leakage of insufflation gas and surgery could proceed quite smoothly. For this phenomenon, we suppose that there might be two reasons as follows: (1) the pressure of thoracic cavity is 10–20 mm Hg with an artificial respirator, whereas the pressure of abdominal cavity is 8–10 mm Hg with insufflation, and (2) since both sides of the crus of diaphragm are preserved, the portion of withdrawal of the esophagus is very narrow. Therefore, the body position needs to be changed only once, because manipulations in the thoracic cavity can be performed prior to the abdominal procedure.

The one of most outstanding advantages in our approach is that dissection of the greater omentum begins at the same height as the inferior edge of the spleen to preserve the right gastroepiploic vessels. In general, the communicating site of the right and left gastroepiploic vessels is slightly caudal to the inferior splenic margin. Accordingly, if the gastroepiploic vessels should be dissected along the greater curvature from slightly further to the cephalic side than the splenic inferior edge, the right gastroepiploic vessels can be completely preserved. Especially for patients with extensive fat in the abdominal cavity, our approach makes it possible to preserve the right gastroepiploic vessels securely, because the point of confluence of the right and left gastroepiploic vessels sometimes cannot be visibly recognized.

Jagot *et al.* reported that the mean time required for gastric conduit construction was 203 minutes by the totally laparoscopic approach [9]. In contrary, Yoshida *et al.* reported that gastric conduit construction by the conventional HALS approach required 275 minutes [22]. In our approach, the time in surgery was 150–165 minutes for the first 3 cases, but wasted 90 minutes for the last 3 cases. This outcome is comparable to the time required for open surgery, and it is believed that with greater experience, the time in surgery can be reduced still further.

The introduction of HALS as an advanced laparoscopic technique has fascilitated the gastric conduit construction. With still more advances and improvements in operative procedures in the future, we believe that endoscopic surgery will be applied to all fields of surgery.

### Acknowledgement

The authors are indebted to Prof. J. Patrick Barron of the International Medical Communications Center of Tokyo Medical University for his review of this manuscript.

### References

[1] Peracchia, A., Ancona, E., Ruol, A. *et al.* Use of mini-invasive procedures in esophageal surgery. *Chirurgie* 1992; **118**: 305–309.
[2] Collard, J.M., Lengele, B., Otte, J.B. *et al.* En bloc and standard esophagectomies by thoracoscopy. *Ann. Thorac. Surg.* 1993; **56**: 675–679.
[3] Gossot, D., Fourquier, P. and Celerier, M. Thoracoscopic esophagectomy: Technique and initials results. *Ann. Thorac. Surg.* 1993; **56**: 667–670.
[4] Liu, H.P., Chang, C.H., Lin, P.J. *et al.* Video-assisted endoscopic esophagectomy with stapled intrathoracic esophagogastric anastomosis. *World J. Surg.* 1995; **19**: 745–747.
[5] Akaishi, T., Kaneda, I., Higuchi, Y. *et al.* Thoracoscopic en bloc total esophagectomy with radical midiastinal lymphadenectomy. *J. Thorac. Cardiovasc. Surg.* 1996; **112**: 1533–1541.

[6] Peracchia, A., Rosati, R., Fumagalli, U. *et al.* Thoracoscopic dissection of the esophagus for cancer. *Int. Surg.* 1997; **82**: 1–4.
[7] Law, S., Fok, M., Chu, K.M. *et al.* Thoracoscopic esophagectomy for esophageal cancer. *Surgery* 1997; **122**: 8–14.
[8] Kawahara, K., Maekawa, T., Okabayashi, K. *et al.* Video-assisted thoracoscopic esophagectomy for esophageal cancer. *Surg. Endosc.* 1999; **13**: 218–223.
[9] Jagot, P., Sauvanet, A., Berthoux, L. *et al.* Laparoscopic mobilization of the stomach for oesophageal replacement. *Br. J. Surg.* 1996; **83**: 540–542.
[10] Nguyen, N.T., Schauer, P.R. and Luketich, J. Combined laparoscopic and thoracoscopic approach to esophagectomy. *J. Am. Coll. Surg.* 1999; **188**: 328–332.
[11] Inoue, H., Izumi, Y., Nagai, K. *et al.* Endoscopic esophagectomy and reconstruction for thoracic esophageal cancer. *Operation* 1998; **52**: 879–884.
[12] Nishihara, T., Mori, S. and Hirayama, K. Extensive lymph node dissection for thoracic esophageal carcinoma. *Dis. Esophagus* 1992; **5**: 79–89.
[13] DePaula, A.L., Hashida, K., Ferreira, E.A.B. *et al.* Laparoscopic transhiatal esophagectomy with esophagogastroplasty. *Surg. Laparosc. Endosc.* 1995; **5**: 1–5.
[14] Swanstrom, L.L. and Hansen, P. Laparoscopic total esophagectomy. *Arch. Surg.* 1997; **132**: 943–949.
[15] Luketich, J.D., Nguyen, N.T., Weigel, T. *et al.* Minimally invasive approach to esophagectomy. *JSLS* 1998; **2**: 243–247.
[16] McMahon, A.J., Russell, I.T., Ramsay, G. *et al.* Laparoscopic and minilaparotomy cholecystectomy: A randomized trial comparing postoperative pain and pulmonary function. *Surgery* 1994; **115**: 533–539.
[17] Coelho, J.C., de Araujo, R.P., Marchesini, J.B. *et al.* Pulmonary function after cholecystectomy performed through Kocher's incision, a mini-incision, and laparoscopy. *World J. Surg.* 1993; **17**: 544–546.
[18] Kusminsky, R.E., Boland, J.P., Tiley, E.H. *et al.* Hand-assisted laparoscopic splenectomy. *Surg. Laparosc. Endosc.* 1995; **5**: 463–467.
[19] Gerhart, C.D. Hand-assisted laparoscopic transhiatal esophagectomy usinf dexterity pneumo sleeve. *JSLS* 1998; **2**: 295–298.
[20] Southern Operator's Club Study Group. Handoscopic Surgery: A prospective multicenter trial of a minimally invasive technique for complex abdominal surgery. *Arch. Surg.* 1999; **134**: 477–485.
[21] The HALS Study Group. Hand-assisted laparoscopic surgery (HALS) with the handport system: Initial experience with 68 patients. *Ann. Surg.* 2000; **231**: 715–723.
[22] Yoshida, T., Takeshita, K., Inoue, H. *et al.* A case of creation of gastric conduit by hand-assisted laparoscopic surgery for esophageal cancer. *JSES* 1998; **3**, 311–315.
[23] Ozawa, S., Ando, N., Ohgami, M. *et al.* Laparoscopic construction of gastric conduit; Less invasive surgery for thoracic esophageal cancer. *Operation* 1999; **53**: 1583–1589.

